# Changes in daily intake of nutrients and foods including confectionery after the initiation of empagliflozin in Japanese patients with type 2 diabetes: a pilot study

**DOI:** 10.1186/s40795-024-00902-5

**Published:** 2024-07-04

**Authors:** Toshiko Murayama, Michihiro Hosojima, Hideyuki Kabasawa, Takahiro Tanaka, Nobutaka Kitamura, Mai Tanaka, Shoji Kuwahara, Yoshiki Suzuki, Ichiei Narita, Akihiko Saito

**Affiliations:** 1 Division of Clinical Nephrology and Rheumatology, Kidney Research Center, Niigata University Graduate School of Medical and Dental Sciences, Niigata City, Niigata, Japan; 2 Department of Health Nutrition, University of Niigata Prefecture Faculty of Human Life Studies, Niigata City, Niigata, Japan; 3 Department of Clinical Nutrition Science, Kidney Research Center, Niigata University Graduate School of Medical and Dental Sciences, 1-757 Asahimachi-dori, Chuo-ku, Niigata City, Niigata, 951-8510 Japan; 4 Clinical and Translational Research Center, Niigata University Medical and Dental Hospital, Niigata City, Niigata, Japan; 5 Department of Applied Molecular Medicine, Kidney Research Center, Niigata University Graduate School of Medical and Dental Sciences, Niigata City, Niigata, Japan; 6 Laboratory of Clinical Nutrition, Department of Nutrition, School of Human Cultures, The University of Shiga Prefecture, Hikone, Shiga Japan

**Keywords:** Empagliflozin, Sodium-glucose cotransporter 2 inhibitors, Dietary intake, Self-administered diet history questionnaire, Type 2 diabetes

## Abstract

**Introduction:**

It is unclear how dietary intake changes after sodium-glucose cotransporter 2 inhibitor (SGLT2i) treatment is started in patients with type 2 diabetes.

**Methods:**

We performed a non-controlled, open-label study that enrolled 51 patients with type 2 diabetes. The patients were newly administered empagliflozin, and their dietary habits were examined using a self-administered diet history questionnaire at the beginning of the study and after 24 weeks. We investigated the association of changes in HbA1c and body weight with changes in energy, nutrient, and food group intakes.

**Results:**

At 24 weeks after the start of the study, HbA1c improved significantly and body weight decreased. In the food group, only the intake of confectionery increased, and there were no significant differences in the association between changes in HbA1c and body weight and changes in energy, nutrient, and food group intakes after 24 weeks. However, a significant negative correlation was found between change in HbA1c after 4 weeks and change in energy intake after 24 weeks, and principal component analysis showed an association between change in HbA1c levels after 4 weeks and change in energy intake and some food group intakes including confectionery after 24 weeks.

**Conclusion:**

In this study, after 24 weeks of treatment with empagliflozin, only intake of confectionery increased. Early assessment by dietitians after initiation of SGLT2i treatment might be important because our data suggested that the reduction in blood glucose levels after the start of empagliflozin was associated with a subsequent increase in energy intake.

**Trial registration:**

University Hospital Medical Information Network-Clinical Trials Registry (UMIN-CTR) on September 5, 2016 (registration ID, UMIN000002309|| http://www.umin.ac.jp/ctr/).

**Supplementary Information:**

The online version contains supplementary material available at 10.1186/s40795-024-00902-5.

## Introduction

Sodium-glucose cotransporter 2 inhibitor (SGLT2i) reduce blood glucose levels by inhibiting reabsorption of glucose in the renal proximal tubules and increasing excretion of glucose in the urine. Urinary excretion of glucose improves glycemic control and induces weight loss if patients maintain the same nutritional intake as before treatment. However, these effects have been reported to be less than expected, considering the increased urinary excretion of glucose [1, 2]. In addition, the degree to which these drugs improve glycemic control and induce weight loss varies among individual patients [3].

SGLT2i-related overeating has been pointed out as one of the causes [4, 5]. Results from animal studies suggest that the effects of SGLT2i on appetite lead to an increase in nutrient intake [6–8]. In clinical practice, it has been speculated that SGLT2i may have some effect on appetite, resulting in altered nutrient intake. An intervention trial with canagliflozin reported an increase in food intake inversely proportional to weight loss [1]. In addition, ipragliflozin treatment was reported to decrease serum leptin levels and increase appetite [9]. In Japan, there are reports of increased sugar intake after starting dapagliflozin [10].

However, a recent study reported that dapagliflozin treatment may not affect food intake or the brain’s response to appetite [11]. Other studies have also found no difference in dietary intake [11–14], and thus the evidence is inconclusive in this regard. Specific changes in intake of different nutrients and food groups and how they relate to the effects of SGLT2i are also unclear. Clarification of these relationships may lead to more effective treatment.

Therefore, in this study, we investigated the relationship of the effects of empagliflozin, an SGLT2i, on glycemic control and weight loss with changes in dietary intake in Japanese patients with type 2 diabetes, using a self-administered diet history questionnaire (DHQ).

## Methods

### Participants

Participants were patients with type 2 diabetes being treated at the Department of Nephrology and Rheumatology at Niigata University Medical and Dental Hospital or an affiliated hospital between December 2016 and February 2018 and satisfied the following inclusion criteria: age 20 to 79 years; glycated hemoglobin (HbA1c) of at least 6.5% and mild or moderate renal dysfunction; able to cope with dehydration and suspend taking medicine appropriately on sick days with fever, diarrhea, vomiting, or appetite loss; and provided written informed consent to participate in the study. The participants received no special guidance regarding lifestyle, including diet and exercise, during the study period, and received standard medical care and support based on the same practice guidelines as before the study started. Exclusion criteria were severe ketosis, diabetic coma or precoma, severe infection, recent or upcoming surgery, severe trauma, debility, severe hepatic impairment, pregnancy or intention to become pregnant, current malignancy or history of malignancy in the past 5 years, and current use of an SGLT2i.

### Study design

This was a single-arm, non-randomized, open-label, uncontrolled interventional study. We prospectively investigated changes in dietary intake and analyzed the effect of food groups on the efficacy of SGLT2i. Patients who consented to participate in this study were treated with oral empagliflozin 10 mg once daily. A researcher held an informed consent discussion with each candidate using written information, and patients who consented to participate reported their dietary intake using a DHQ before the start of treatment with empagliflozin and at 24 weeks after the start of treatment. Because this was a pilot study and the data necessary to calculate the optimal sample size were not available in advance, the sample size was determined to be around 50 cases, the maximum number for which the research team could conduct a study including the DHQ. The study design conformed to the principles of the Declaration of Helsinki, was approved by the institutional review board of Niigata University (Approval No. 2550), and was registered in the UMIN-CTR clinical trial database (UMIN000023902).

### Measurements

In this study, the DHQ was used to assess dietary intake. The DHQ makes it possible to estimate the intake of about 150 types of food and beverage items, energy intake, nutrient intake, and intake of food groups [15]. The DHQ survey, which is answered mainly by a mark-sheet system, investigates eating habits over the previous month. Participants are asked to self-report over 400 items, including eating habits, frequency of food intake, cooking methods, and staple-food amounts. Based on the answers, a special calculation program using the Japanese Standard Tables of Composition [16] was used to calculate the intake of nutrients [15, 17]. The validity of the DHQ has been verified through comparison with other measures such as 3-day dietary records, 24-h urine collection, serum biomarkers, and doubly labeled water [15, 17–20]. The DHQ form was distributed to patients before the start of treatment and again at 24 weeks after the start of treatment. All patients completed the form independently during an office visit or at home. A registered dietician reviewed the forms for errors, and, upon finding a blank answer or illogical response, asked the patient for the correct response and recorded it.

Parameters analyzed to assess the effects of empagliflozin were body weight, body mass index (BMI), and biochemical tests. Values such as body weight and HbA1c were measured at baseline and at 4, 12, and 24 weeks after the start of treatment with empagliflozin. BMI was calculated by dividing body weight (kg) by the square of height (m). Standard weight was defined according to the formula 22 × height (m) × height (m). The estimated glomerular filtration rate (eGFR) was calculated as 194 × serum creatinine (Cr) − 1.094 × age − 0.287 for men and 194 × serum Cr − 1.094 × age − 0.287 × 0.739 for women [21]. Serum Cr was measured using the enzymatic assay method.

### Statistical analyses

All statistical analyses were performed by T.T. and N.K., both of whom are statisticians. The results are presented as means ± SD or numbers (percentages). Data on daily energy intake, nutrient intake, and intake of food groups obtained through the DHQ were used in the analysis. To examine changes before and after empagliflozin administration, intake before and after initiation was compared using a paired *t*-test.

To examine the relationship of changes in HbA1c and body weight with compensatory overeating associated with empagliflozin treatment, we also examined the correlation of changes in HbA1c and body weight at each of 4, 12, and 24 weeks with changes in energy, carbohydrate, protein, fat, and food group intake at 24 weeks, using Pearson’s correlation coefficient. In addition, principal component analysis was performed using the 4-week change in HbA1c and the 24-week change in energy and food group intake as variables to examine the associations between these variables. Eigenvalues, contribution ratios, and cumulative contribution ratios for each principal component were obtained from the principal component analysis. Factor loadings for each variable were also obtained, and scatter plots of the factor loadings were plotted for each variable.

All statistical analyses were performed using SAS Statistics ver. 9.4 (SAS Institute Inc., Cary, NC). All statistical tests were two-sided, and differences were considered statistically significant when the probability of the alpha error was less than 5%.

## Results

Fifty-one patients consented to participate in this study, and all were included in our analysis. Two patients with skin disorders or liver dysfunction discontinued treatment with empagliflozin, so there were no blood tests or other data available for them after 4 weeks. In addition, there were two other patients who could not come to the hospital within the allowance of the 24-week visit (Figure S1).

Table 1 shows the clinical characteristics of participants at baseline and 24 weeks. The average age was 62.4 ± 11.2 years. After 24 weeks of treatment with empagliflozin, significant decreases were noted in weight (from 72.7 ± 16.7 kg to 69.6 ± 17.0 kg, *p* < 0.001), BMI (from 27.8 ± 5.0 kg/m^2^ to 26.7 ± 5.2 kg/m^2^, *p* < 0.001), and eGFR (from 65.1 ± 17.9 mL/min/1.73 m^2^ to 62.8 ± 17.8 mL/min/1.73 m^2^, *p* = 0.002), and significant improvement was noted in HbA1c, with a reduction from 7.6% ± 1.2% to 7.1% ± 1.0% (*p* < 0.001). Table 2(a) shows changes in nutrient intake after 24 weeks of treatment with empagliflozin. Energy and carbohydrate intakes did not differ significantly, increasing from 1846 ± 554 to 1889 ± 504 kcal/day (*p* = 0.263) and from 244.7 ± 80.2 to 257.7 ± 69.4 g/day (*p* = 0.134), respectively. There was no significant difference in the intakes of 9 vitamins and 8 minerals (data not shown). Table 2(b) shows the changes in food group intakes after 24 weeks of treatment with empagliflozin. Confectionery intake tended to increase from 53.6 ± 46.5 g to 68.5 ± 57.8 g (*p* = 0.021). No significant differences were observed for any other food groups.

**Table 1:** Clinical characteristics of participants at baseline and at 24 weeks

		Baseline			24 weeks		*p*-value^†^	
Sex (Male: Female)		32:19						
Age	years	62.4 ± 10.7						
Body weight	kg	72.7 ± 16.7			69.6 ± 17.0		< 0.001	
BMI	kg/m^2^	27.8 ± 5.0			26.7 ± 5.2		< 0.001	
HbA1c	%	7.6 ± 1.2			7.1 ± 1.0		< 0.001	
eGFR	mL/min/1.73m^2^	65.1 ± 17.9			62.8 ± 17.8		0.002	
Pharmacological treatment,								
Insulin	*n* (%)	9(17.6)			7 (13.7)			
GLP-1 receptor agonist	*n* (%)	1 (2.0)			1 (2.0)			
DPP-4 inhibitor	*n* (%)	39 (76.5)			36 (70.5)			
Sulfonylurea	*n* (%)	7 (13.7)			6 (11.8)			
Glinide	*n* (%)	7 (13.7)			7 (13.7)			
Biguanide	*n* (%)	20 (39.2)			20 (39.2)			
α-glucosidase inhibitor	*n* (%)	13 (25.5)			12 (23.5)			
Thiazolidinedione	*n* (%)	7 (13.7)			7 (13.7)			

Data are expressed as the mean ± standard deviation or number (percentage)

*BMI* body mass index, *Cr* creatinine, *eGFR* estimated glomerular filtration rate, *GLP* glucagon-like peptide, *DPP* dipeptidyl peptidase,^†^Paired *t*-test

**Table 2:** Comparisons of energy, nutrient, and food group intakes at baseline and at 24 weeks

(a)			
Energy and nutrients	Baseline	24 weeks	*p*-value^†^
Energy (kcal/day)	1846 ± 554	1889 ± 504	0.263
Carbohydrate (g/day)	244.7 ± 80.2	257.7 ± 69.4	0.134
Protein (g/day)	66.6 ± 22.6	63.7 ± 18.3	0.532
Fat (g/day)	56.0 ± 23.6	55.4 ± 25.3	0.926
(b)			
Food groups	Baseline (g/day)	24 weeks (g/day)	*p*-value^†^
Cereals	413.6 ± 180.6	426.9 ± 184.6	0.434
Potatoes	23.5 ± 19.1	24.1 ± 15.9	0.879
Sugars	9.5 ± 5.5	9.3 ± 5.8	1.000
Pulses	51.9 ± 37.4	56.8 ± 45.5	0.281
Nuts	3.2 ± 6.5	2.0 ± 3.5	0.152
Green and yellow vegetables	94.3 ± 66.9	95.6 ± 74.4	0.964
Other vegetables	118.2 ± 81.8	113.3 ± 65.0	0.526
Fruits	92.2 ± 71.2	146.3 ± 209.0	0.086
Mushrooms	11.1 ± 17.2	7.8 ± 10.5	0.095
Seaweeds	8.2 ± 7.6	9.6 ± 11.0	0.262
Fish and shellfish	78.6 ± 53.4	67.8 ± 36.1	0.232
Meats	63.3 ± 41.4	56.4 ± 37.1	0.225
Eggs	40.4 ± 30.4	40.5 ± 33.3	0.815
Dairy products	107.7 ± 118.4	106.8 ± 109.3	0.865
Animal fats	0.5 ± 1.5	0.5 ± 1.0	0.710
Vegetable oils	20.4 ± 14.4	20.5 ± 17.5	0.788
Confectioneries	53.6 ± 46.5	68.5 ± 57.8	0.021
Alcoholic beverages	89.2 ± 183.5	104.5 ± 178.8	0.094
Non-alcoholic beverages	868.9 ± 637.2	908.1 ± 561.1	0.821
Salt-based seasonings	15.5 ± 12.7	13.3 ± 8.4	0.071

Data are expressed as the mean ± standard deviation. †, Paired *t*-test

**Fig. 1:**
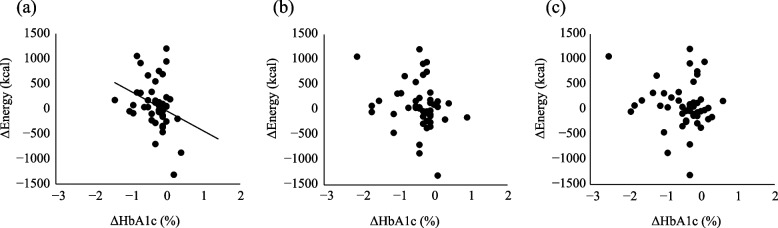
Correlation analysis between changes in HbA1c and energy intake from baseline to 24 weeks

The changes from baseline in HbA1c and body weight at 4, 12, and 24 weeks were − 0.3% ± 0.3%, − 0.4% ± 0.5%, and − 0.5% ± 0.6% for HbA1c and − 1.3 ± 1.1 kg, − 2.0 ± 1.8 kg, and − 2.7 ± 2.5 kg for body weight, respectively. Although the amount of change became progressively smaller, each significantly decreased from baseline (Table S1). Correlations of changes in HbA1c and body weight with changes in energy, carbohydrate, protein, fat, and food group intake after 24 weeks were examined (Fig. 1, Table S2, S3). No significant relationships were found between change in HbA1c or body weight after 24 weeks and change in energy intake after 24 weeks. However, a significant negative correlation was found between change in HbA1c after 4 weeks and change in energy after 24 weeks. Also, the magnitudes of the correlation coefficients between the change in HbA1c at each period and energy intake after 24 weeks were in the following order, from highest to lowest: 4 weeks, 12 weeks, and 24 weeks. These findings indicate that the greater the decrease in early HbA1c, the greater the final energy intake after 24 weeks. No significant relationship with body weight was found.

**Fig. 2:**
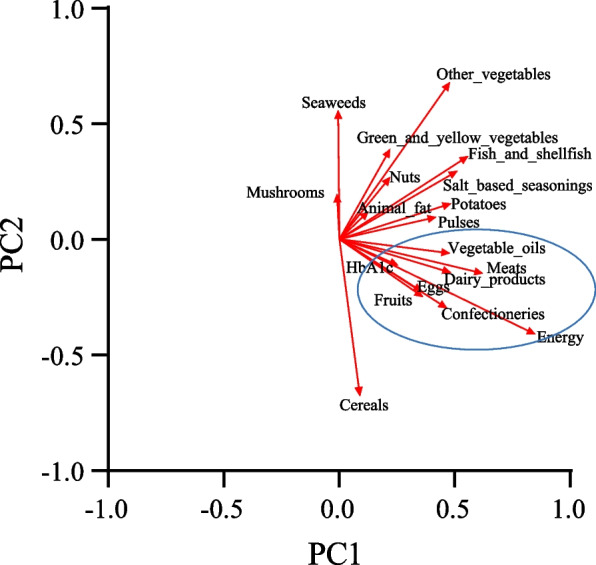
Principal component analysis results by change in HbA1c after 4 weeks and change in energy, nutrient, and foods group intake after 24 weeks

Positive correlations between change in energy intake and change in food group intake were found for several food groups, among which the correlation coefficient for change in energy and confectionery intake had the highest value (*r* = 0.496, *p* < 0.001) (Table S4). Because a significant correlation was found between change in HbA1c at 4 weeks and change in energy at 24 weeks, further analysis was performed, using scatter plots of factor loadings for the first and second principal components of the principal component analysis with HbA1c change at 4 weeks as well as energy and food group intake at 24 weeks as variables. The results showed that the same groups were identified for change in HbA1c as well as energy, confectionery, meat, dairy products, vegetable fats and oils, eggs, and fruit (Fig. 2, groups enclosed by blue lines, Table S5).

## Discussion

This study examined changes in dietary intake after 24 weeks of treatment with empagliflozin in patients with type 2 diabetes and investigated the association of changes in HbA1c and body weight with changes in energy, nutrient, and food group intakes. After 24 weeks, significant increases were found only in confectionery intake. A significant negative correlation between change in energy intake after 24 weeks and change in HbA1c level after 4 weeks suggested that energy intake increases more when the reduction in blood glucose is greater at the early stage of treatment, which was also associated with food groups such as confectionery. No obvious SGLT2i-related overeating was observed in the overall participant population but it might have occurred in a subpopulation.

Our data showed that a greater reduction in blood glucose levels very early in the start of empagliflozin treatment may lead to an increase in subsequent energy intake. It is well known that a reduction in blood glucose triggers the sensation of hunger and stimulates appetite [22]. A study of healthy individuals showed the possibility that neurons in the brain responded more strongly to visual simulation of high-calorie foods, and consequently, there was a higher risk of overeating in those with mild hypoglycemia than in those with normoglycemia [23]. In addition, in a study of patients with obesity and type 2 diabetes that demonstrated dapagliflozin’s effect on the central nerve system (including suppressed responses to visual simulation of high-calorie foods), appetite for high-calorie foods was increased after short-term treatment [24].

A previous study of SGLT2i and confectionery intake in healthy individuals showed that the postprandial desire for sweets tended to be higher, albeit with no significant increases in energy intake, after 2-week treatment with dapagliflozin [25]. Meanwhile, increased sugar intake 12 weeks after initiation of treatment with dapagliflozin has also been reported [10]. In addition, HbA1c levels did not decrease after initiation of luseogliflozin in patients with a habit of nighttime snacking, indicating that the effect of the agent had attenuated [26]. Furthermore, an epidemiological study of patients with type 2 diabetes based on health checkup data showed that having unfavorable eating habits such as eating snacks after the evening meal was associated with poor glycemic control and obesity, even during treatment with antidiabetics such as SGLT2i [27]. As mentioned above, prolonged increases in confectionery intake during SGLT2i treatment may attenuate the effect of the agent. In the present study, an increase in confectionery intake was observed in the before-and-after comparison. Moreover, our principal component analysis, which examined the relationships of changes in HbA1c level after 4 weeks with changes in food group intakes after 24 weeks, showed associations of changes in HbA1c level with changes in energy intake and confectionery intake. Significant changes in confectionery intake after initiation of SGLT2i treatment indicates the possibility that appetite was increased in patients with marked decreases in blood glucose level at the early stage of treatment, leading to increases in the intake of confectioneries, which are characterized by their high calories and easy availability.

In this study, no significant differences were found in the association between changes in dietary intake and the effect of reducing blood glucose and body weight at initiation vs. after 24 weeks. However, there have been reports of a tendency toward SGLT2i-related overeating in trials with a duration of less than 24 weeks [2, 10]. Miura et al. reported that ipragliflozin decreased the serum leptin level, which possibly resulted in increased appetite 2–8 weeks after administration, although appetite reverted to baseline 16 weeks after initiation of treatment, albeit with persistently low levels of serum leptin [9]. A study of patients with obesity and type 2 diabetes who were administered dapagliflozin reported that brain activation in response to the anticipation of food intake increased after 10 days, but not after 16 weeks, suggesting that dapagliflozin may cause overeating for a short period after administration [28]. Taken together, SGLT2i-related overeating may last for a relatively short period of time. It is possible that SGLT2i-related overeating might have been observed in the present study if the dietary survey had been conducted earlier than 24 weeks after initiation of treatment.

Another possible consideration for the above is the effect of the anti-diabetic drugs the patients were using. Ferrannini et al. [2] and Horie et al. [10] speculated that SGLT2i-related overeating may not be obvious if a large proportion of the study population is being treated with oral agents such as metformin and GLP-1 receptor agonists that suppress appetite. Postprandial decreases in ghrelin level lasted longer in patients treated with metformin than in those treated with diet alone, and in the appetite evaluation, there was an association between the sensation of fullness and the suppression of hunger in patients treated with metformin, indicating possible decreases in energy intake [29]. In a recent study finding that energy intake did not increase [14], approximately 50% of patients were treated with biguanides (including metformin). In the present study, approximately 40% of participants were treated with metformin, which might explain why energy intake did not change.

A study of 24-week empagliflozin treatment with nutritional education showed significant decreases in body weight and improvement in blood glucose level without postprandial hunger sensation in the empagliflozin-treated group compared with the placebo group [30]. A 12-month observational study of dapagliflozin in combination with dietary carbohydrate restriction and intervention by specialist weight-management dietitians throughout the study period showed decreases in body weight and blood leptin level but no changes in appetite in patients with obesity and type 2 diabetes [31]. Taken together, previous research suggests that administration of oral SGLT2i combined with nutritional education may be an effective treatment that suppresses appetite. The present study showed that confectionery intake increased after 24 weeks and that lower blood glucose levels in the early treatment period may be associated with overeating after 24 weeks, indicating the need for nutritional education in such cases.

The limitations of this study include its small sample size, the fact that it was an open-label unblinded study, and lack of a control group, and thus more evidence-based research based on this study is warranted. Another limitation is that changes in the intake of different nutrients and food groups were examined over only 24 weeks of treatment with empagliflozin. Changes would need to be examined for longer than 24 weeks if treatment were to be continued. Although the participants did not receive any specific instructions regarding diet, exercise, or other lifestyle habits, it is possible that some might have intentionally changed their lifestyle during the study period. It has been reported that diet varies according to the season [32], but this was not taken into account in the present study. We also did not examine subjective symptoms such as hunger and appetite. Future studies will need to examine how signs of SGLT2i-related overeating, including hunger, appetite, changes in taste and preferences, thirst level, and frequency of eating between meals, relate to dietary intake. Because the participants were able to check changes in HbA1c and weight during the examination, it is possible that they subconsciously attempted to adjust aspects of their lifestyle after learning of these changes. It is also possible that the second time they took the DHQ, due to their familiarity with the subject, they might have subconsciously given more balanced answers in an attempt to provide positive answers. Because underestimation has been reported [18] and there is a possibility of input errors, the dietitian in this study carefully explained the DHQ procedure at the time of response and checked for input errors when collecting the survey forms. In addition, 6 participants aged 75 years and older were included, and the participants overall had varying baseline renal function (29 participants with eGFR 60 mL/min/1.73 m^2^ or above, 14 participants with 45 to 60, and 6 participants with 30 to 45). Furthermore, in this study, body composition and physical activity could not be investigated. Despite these limitations, this study provides valuable information about changes in dietary intake after the initiation of SGLT2i, including the effects of food group intake on blood glucose and body weight as well as energy and nutrients.

## Conclusion

In this study, after 24 weeks of empagliflozin administration, only intake of confectionery increased and no differences in other food intakes were observed. No change in dietary intake was found to influence the effect of reducing blood glucose and body weight in the before-and-after comparison. However, our data suggested that the reduction in blood glucose levels early after the start of empagliflozin was associated with the subsequent increase in energy intake. Therefore, early assessment by dietitians after initiation of SGLT2i treatment might be important. Further investigation is needed to elucidate the effects of changes in dietary intake early after starting SGLT2 inhibitors.

### Supplementary Information

Supplementary Material 1.

Supplementary Material 2.

Supplementary Material 3.

Supplementary Material 4.

Supplementary Material 5. 

Supplementary Material 6.

## Data Availability

All data generated or analyzed during this study are included in this article.

## Abbreviations

SGLT2i: Sodium-glucose cotransporter 2 inhibitors
DHQ: Diet history questionnaire
BMI: Body mass index
eGFR: Estimated glomerular filtration rate

## References

[1] Polidori D, Sanghvi A, Seeley RJ, Hall KD (2016). How Strongly does appetite counter weight loss? Quantification of the feedback control of human energy intake. Obesity (Silver Spring).

[2] Ferrannini G, Hach T, Crowe S, Sanghvi A, Hall KD, Ferrannini E (2015). Energy balance after sodium-glucose cotransporter 2 inhibition. Diabetes Care.

[3] Kim H, Lee SH, Lee H, Yim HW, Cho JH, Yoon KH (2021). Blood glucose levels and bodyweight change after dapagliflozin administration. J Diabetes Investig.

[4] Blüher M (2022). GLP1 receptor agonist overcomes SGLT2 inhibitor-related overeating. Nat Rev Endocrinol.

[5] Matsuba I, Kanamori A, Takihata M, Takai M, Maeda H, Kubota A (2020). Canagliflozin increases calorie intake in type 2 diabetes without changing the energy ratio of the three macronutrients: CANA-K study. Diabetes Technol Ther.

[6] Devenny JJ, Godonis HE, Harvey SJ, Rooney S, Cullen MJ, Pelleymounter MA (2012). Weight loss induced by chronic dapagliflozin treatment is attenuated by compensatory hyperphagia in diet-induced obese (DIO) rats. Obesity (Silver Spring).

[7] Iuchi H, Sakamoto M, Matsutani D, Suzuki H, Kayama Y, Takeda N (2017). Time-dependent effects of ipragliflozin on behaviour and energy homeostasis in normal and type 2 diabetic rats: continuous glucose telemetry analysis. Sci Rep.

[8] Hashiuchi E, Watanabe H, Kimura K, Matsumoto M, Inoue H, Inaba Y (2021). Diet intake control is indispensable for the gluconeogenic response to sodium-glucose cotransporter 2 inhibition in male mice. J Diabetes Investig.

[9] Miura H, Sakaguchi K, Okada Y, Yamada T, Otowa-Suematsu N, So A (2019). Effects of ipragliflozin on glycemic control, appetite and its related hormones: a prospective, multicenter, open-label study (SOAR-KOBE Study). J Diabetes Investig.

[10] Horie I, Abiru N, Hongo R, Nakamura T, Ito A, Haraguchi A (2018). Increased sugar intake as a form of compensatory hyperphagia in patients with type 2 diabetes under dapagliflozin treatment. Diabetes Res Clin Pract.

[11] Rajeev SP, Roberts CA, Brown E, Sprung VS, Harrold JA, Halford JCG (2023). No evidence of compensatory changes in energy balance, despite reductions in body weight and liver fat, during dapagliflozin treatment in type 2 diabetes mellitus: a randomized, double-blind, placebo-controlled, cross-over trial (ENERGIZE). Diabetes Obes Metab.

[12] Tahara A, Kondo Y, Takasu T, Tomiyama H (2018). Effects of the SGLT2 inhibitor ipragliflozin on food intake, appetite-regulating hormones, and arteriovenous differences in postprandial glucose levels in type 2 diabetic rats. Biomed Pharmacother.

[13] Kosugi R, Nakatani E, Okamoto K, Aoshima S, Arai H, Inoue T (2019). Effects of sodium-glucose cotransporter 2 inhibitor (dapagliflozin) on food intake and plasma fibroblast growth factor 21 levels in type 2 diabetes patients. Endocr J.

[14] Yabe D, Shiki K, Homma G, Meinicke T, Ogura Y, Seino Y (2023). Efficacy and safety of the sodium-glucose co-transporter-2 inhibitor empagliflozin in elderly Japanese adults (≥65 years) with type 2 diabetes: a randomized, double-blind, placebo-controlled, 52-week clinical trial (EMPA-ELDERLY). Diabetes Obes Metab.

[15] Sasaki S, Yanagibori R, Amano K (1998). Validity of a self-administered diet history questionnaire for assessment of sodium and potassium: comparison with single 24-hour urinary excretion. Jpn Circ J.

[16] Science and Technology Agency (2005). Standard tables of food composition in Japan, fifth revised and enlarged edition (in Japanese).

[17] Sasaki S, Ushio F, Amano K, Morihara M, Todoriki O, Uehara Y (2000). Serum biomarker-based validation of a self-administered diet history questionnaire for Japanese subjects. J Nutr Sci Vitaminol (Tokyo).

[18] Okubo H, Sasaki S, Rafamantanantsoa HH, Ishikawa-Takata K, Okazaki H, Tabata I (2008). Validation of self-reported energy intake by a self-administered diet history questionnaire using the doubly labeled water method in 140 Japanese adults. Eur J Clin Nutr.

[19] Kobayashi S, Murakami K, Sasaki S, Okubo H, Hirota N, Notsu A (2011). Comparison of relative validity of food group intakes estimated by comprehensive and brief-type self-administered diet history questionnaires against 16 d dietary records in Japanese adults. Public Health Nutr.

[20] Kobayashi S, Honda S, Murakami K, Sasaki S, Okubo H, Hirota N (2012). Both comprehensive and brief self-administered diet history questionnaires satisfactorily rank nutrient intakes in Japanese adults. J Epidemiol.

[21] Matsuo S, Imai E, Horio M, Yasuda Y, Tomita K, Nitta K (2009). Revised equations for estimated GFR from serum creatinine in Japan. Am J Kidney Dis.

[22] Mayer J (1953). Glucostatic mechanism of regulation of food intake. N Engl J Med.

[23] Page KA, Seo D, Belfort-DeAguiar R, Lacadie C, Dzuira J, Naik S (2011). Circulating glucose levels modulate neural control of desire for high-calorie foods in humans. J Clin Invest.

[24] van Ruiten CC, Veltman DJ, Schrantee A, van Bloemendaal L, Barkhof F, Kramer MHH (2022). Effects of dapagliflozin and combination therapy with exenatide on food-cue induced brain activation in patients with type 2 diabetes. J Clin Endocrinol Metab.

[25] Bertran E, Berlie HD, Nixon A, Jaber L (2019). Does dapagliflozin affect energy intake and appetite? A randomized, controlled exploratory study in healthy subjects. Clin Pharmacol Drug Dev.

[26] Furukawa S, Miyake T, Miyaoka H, Matsuura B, Hiasa Y (2022). Observational study on unhealthy eating behavior and the effect of sodium-glucose cotransporter 2 inhibitors: the luseogliflozin ehime diabetes study. Diabetes Ther.

[27] Gouda M, Matsukawa M, Iijima H (2018). Associations between eating habits and glycemic control and obesity in Japanese workers with type 2 diabetes mellitus. Diabetes Metab Syndr Obes.

[28] van Ruiten CC, Veltman DJ, Wijdeveld M, Ten Kulve JS, Kramer MHH, Nieuwdorp M (2022). Combination therapy with exenatide decreases the dapagliflozin-induced changes in brain responses to anticipation and consumption of palatable food in patients with type 2 diabetes: a randomized controlled trial. Diabetes Obes Metab.

[29] English PJ, Ashcroft A, Patterson M, Dovey TM, Halford JC, Harrison J (2007). Metformin prolongs the postprandial fall in plasma ghrelin concentrations in type 2 diabetes. Diabetes Metab Res Rev.

[30] Sargeant JA, King JA, Yates T, Redman EL, Bodicoat DH, Chatterjee S (2022). The effects of empagliflozin, dietary energy restriction, or both on appetite-regulatory gut peptides in individuals with type 2 diabetes and overweight or obesity: the SEESAW randomized, double-blind, placebo-controlled trial. Diabetes Obes Metab.

[31] Hanson P, Randeva H, Cuthbertson DJ, O'Hare PJ, Parsons N, Chatha K (2022). The DAPA-DIET study: metabolic response to dapagliflozin combined with dietary carbohydrate restriction in patients with type 2 diabetes mellitus and obesity-a longitudinal cohort study. Endocrinol Diabetes Metab.

[32] Tokudome Y, Imaeda N, Nagaya T, Ikeda M, Fujiwara N, Sato J (2002). Daily, weekly, seasonal, within- and between-individual variation in nutrient intake according to four season consecutive 7 day weighed diet records in Japanese female dietitians. J Epidemiol.

