# Supplementary figures and images for: Changes in daily intake of nutrients and foods including confectionery after the initiation of empagliflozin in Japanese patients with type 2 diabetes: a pilot study
Source: BMC Nutr. 2024 Jul 4;10:95. doi: 10.1186/s40795-024-00902-5 (PMC11229015; doi:10.1186/s40795-024-00902-5)

Figure S1.

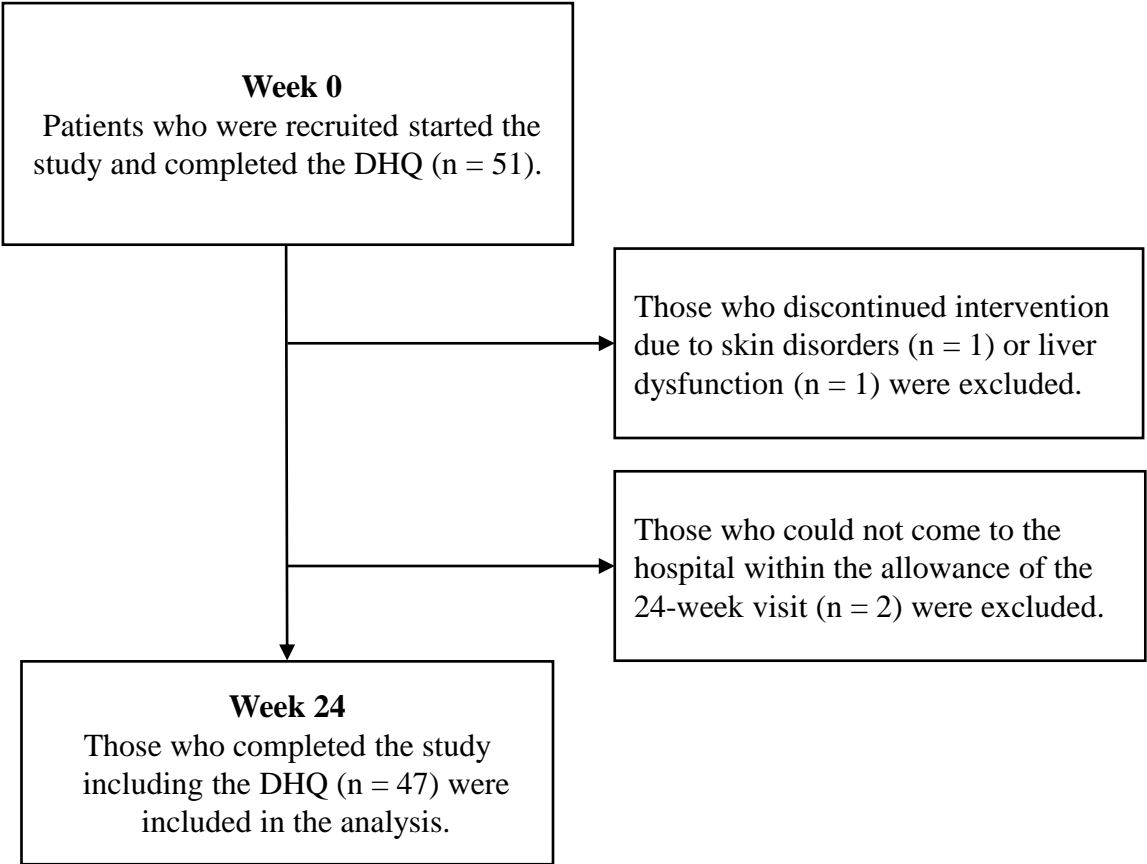

Supplement: Supplementary file 1 — Supplementary Material 1. [file 40795_2024_902_MOESM1_ESM.pdf]
