# Supplementary material for: Changes in daily intake of nutrients and foods including confectionery after the initiation of empagliflozin in Japanese patients with type 2 diabetes: a pilot study
Source: BMC Nutr. 2024 Jul 4;10:95. doi: 10.1186/s40795-024-00902-5 (PMC11229015; doi:10.1186/s40795-024-00902-5)
Supplement: Supplementary file 2 — Supplementary Material 2. [file 40795_2024_902_MOESM2_ESM.docx]

**Table S1.** Changes in HbA1c and body weight after 4, 12, and 24 weeks

|  |  | Baseline | | | 4 weeks | | | 12 weeks | | | 24 weeks | | |
| --- | --- | --- | --- | --- | --- | --- | --- | --- | --- | --- | --- | --- | --- |
| HbA1c (%) | | 7.6 | ± | 1.2 | 7.3 | ± | 1.1 | 7.2 | ± | 1.1 | 7.1 | ± | 1.0 |
| Chang from baseline | |  | - |  | −0.3 | ± | 0.3 | −0.4 | ± | 0.5 | −0.5 | ± | 0.6 |
| *p*-value^†^ | |  | | | < 0.001 | | | < 0.001 | | | < 0.001 | | |
| Body weight (kg) | | 72.7 | ± | 16.7 | 70.6 | ± | 15.9 | 69.8 | ± | 16.1 | 69.6 | ± | 17.0 |
| Chang from baseline | |  | - |  | −1.3 | ± | 1.1 | −2.0 | ± | 1.8 | −2.7 | ± | 2.5 |
| *p*-value^†^ | |  | | | < 0.001 | | | < 0.001 | | | < 0.001 | | |

Data are expressed as the mean ± standard deviation or number.

^†^ Paired *t*-test. *p*-value for comparison with baseline.
