# Supplementary material for: Changes in daily intake of nutrients and foods including confectionery after the initiation of empagliflozin in Japanese patients with type 2 diabetes: a pilot study
Source: BMC Nutr. 2024 Jul 4;10:95. doi: 10.1186/s40795-024-00902-5 (PMC11229015; doi:10.1186/s40795-024-00902-5)
Supplement: Supplementary file 3 — Supplementary Material 3. [file 40795_2024_902_MOESM3_ESM.docx]

Table S2. Correlation analysis comparing changes in HbA1c at 4, 12, and 24 weeks with changes in energy, nutrient, and food groups intake after 24 weeks

|  | Changes in HbA1c | | | | | |
| --- | --- | --- | --- | --- | --- | --- |
|  | after 4 weeks | | after 12 weeks | | after 24 weeks | |
|  | *r* | *p-value* | *r* | *p-value* | *r* | *p*-value |
| Δ Energy | −0.302 | 0.039 | −0.237 | 0.108 | −0.148 | 0.320 |
| Δ Carbohydrate | −0.221 | 0.135 | −0.102 | 0.493 | −0.058 | 0.697 |
| Δ Protein | −0.272 | 0.073 | −0.302 | 0.039 | −0.271 | 0.066 |
| Δ Fat | −0.166 | 0.265 | −0.124 | 0.405 | −0.095 | 0.525 |
| Δ Cereals | −0.231 | 0.118 | −0.204 | 0.170 | −0.201 | 0.175 |
| Δ Potatoes | −0.015 | 0.920 | −0.016 | 0.916 | 0.117 | 0.433 |
| Δ Sugars | −0.155 | 0.300 | −0.113 | 0.450 | 0.069 | 0.644 |
| Δ Pulses | −0.171 | 0.250 | −0.126 | 0.398 | −0.073 | 0.626 |
| Δ Nuts | −0.051 | 0.732 | −0.153 | 0.304 | −0.139 | 0.350 |
| Δ Green and yellow vegetables | 0.050 | 0.736 | 0.275 | 0.061 | 0.149 | 0.316 |
| Δ Other vegetables | −0.058 | 0.698 | 0.058 | 0.701 | 0.016 | 0.916 |
| Δ Fruits | 0.136 | 0.362 | −0.175 | 0.240 | 0.161 | 0.278 |
| Δ Mushrooms | −0.185 | 0.212 | −0.069 | 0.645 | −0.047 | 0.756 |
| Δ Seaweeds | 0.033 | 0.826 | 0.186 | 0.210 | 0.125 | 0.403 |
| Δ Fish and shellfish | −0.307 | 0.036 | −0.266 | 0.070 | −0.147 | 0.324 |
| Δ Meats | −0.046 | 0.757 | −0.127 | 0.394 | −0.235 | 0.112 |
| Δ Eggs | −0.146 | 0.328 | −0.080 | 0.592 | −0.119 | 0.426 |
| Δ Dairy products | −0.162 | 0.275 | −0.125 | 0.401 | −0.181 | 0.222 |
| Δ Animal fats | 0.107 | 0.475 | 0.141 | 0.344 | 0.036 | 0.808 |
| Δ Vegetable oils | 0.018 | 0.904 | 0.015 | 0.919 | 0.066 | 0.658 |
| Δ Confectioneries | 0.038 | 0.801 | 0.125 | 0.401 | 0.146 | 0.326 |
| Δ Alcoholic beverages | −0.290 | 0.048 | −0.225 | 0.129 | −0.114 | 0.447 |
| Δ Non-alcoholic beverages | 0.005 | 0.975 | 0.027 | 0.855 | −0.011 | 0.943 |
| Δ Salt-based seasonings | 0.011 | 0.940 | 0.079 | 0.600 | −0.025 | 0.868 |

*r*, Pearson’s correlation coefficient
