# Supplementary material for: Changes in daily intake of nutrients and foods including confectionery after the initiation of empagliflozin in Japanese patients with type 2 diabetes: a pilot study
Source: BMC Nutr. 2024 Jul 4;10:95. doi: 10.1186/s40795-024-00902-5 (PMC11229015; doi:10.1186/s40795-024-00902-5)
Supplement: Supplementary file 4 — Supplementary Material 4. [file 40795_2024_902_MOESM4_ESM.docx]

Table S3. Correlation analysis comparing changes in body weight after 4, 12, and 24 weeks with changes in energy, nutrient, and food groups intake after 24 weeks

|  | Changes in body weight | | | | | |
| --- | --- | --- | --- | --- | --- | --- |
|  | after 4 weeks | | after 12 weeks | | after 24 weeks | |
|  | *r* | *p-value* | *r* | *p-value* | *r* | *p*-value |
| Δ Energy | −0.101 | 0.506 | −0.196 | 0.191 | 0.039 | 0.794 |
| Δ Carbohydrate | 0.056 | 0.711 | −0.161 | 0.285 | 0.043 | 0.775 |
| Δ Protein | −0.081 | 0.593 | −0.132 | 0.383 | 0.016 | 0.915 |
| Δ Fat | −0.188 | 0.210 | −0.144 | 0.341 | −0.059 | 0.694 |
| Δ Cereals | 0.057 | 0.705 | −0.266 | 0.074 | −0.083 | 0.578 |
| Δ Potatoes | −0.093 | 0.538 | 0.078 | 0.605 | 0.241 | 0.103 |
| Δ Sugars | −0.092 | 0.545 | 0.161 | 0.284 | 0.265 | 0.072 |
| Δ Pulses | 0.234 | 0.117 | 0.186 | 0.215 | 0.143 | 0.336 |
| Δ Nuts | −0.260 | 0.081 | −0.274 | 0.065 | −0.262 | 0.075 |
| Δ Green and yellow vegetables | 0.360 | 0.014 | 0.444 | 0.002 | 0.238 | 0.108 |
| Δ Other vegetables | −0.078 | 0.606 | 0.164 | 0.276 | 0.047 | 0.755 |
| Δ Fruits | 0.029 | 0.849 | −0.045 | 0.765 | 0.086 | 0.566 |
| Δ Mushrooms | −0.075 | 0.620 | −0.074 | 0.626 | 0.023 | 0.879 |
| Δ Seaweeds | −0.109 | 0.470 | 0.024 | 0.875 | −0.109 | 0.468 |
| Δ Fish and shellfish | −0.131 | 0.387 | −0.018 | 0.904 | 0.108 | 0.470 |
| Δ Meats | −0.075 | 0.622 | −0.072 | 0.633 | 0.008 | 0.955 |
| Δ Eggs | 0.049 | 0.748 | 0.024 | 0.876 | −0.038 | 0.800 |
| Δ Dairy products | 0.113 | 0.455 | 0.051 | 0.736 | 0.069 | 0.646 |
| Δ Animal fats | 0.101 | 0.504 | −0.021 | 0.891 | −0.074 | 0.623 |
| Δ Vegetable oils | −0.195 | 0.194 | −0.117 | 0.439 | −0.087 | 0.562 |
| Δ Confectioneries | −0.017 | 0.911 | 0.117 | 0.437 | 0.161 | 0.281 |
| Δ Alcoholic beverages | −0.228 | 0.127 | −0.036 | 0.815 | 0.110 | 0.463 |
| Δ Non-alcoholic beverages | 0.052 | 0.730 | −0.071 | 0.637 | −0.140 | 0.349 |
| Δ Salt-based seasonings | −0.211 | 0.158 | −0.050 | 0.741 | −0.069 | 0.644 |

*r*, Pearson’s correlation coefficient
