# Supplementary material for: Changes in daily intake of nutrients and foods including confectionery after the initiation of empagliflozin in Japanese patients with type 2 diabetes: a pilot study
Source: BMC Nutr. 2024 Jul 4;10:95. doi: 10.1186/s40795-024-00902-5 (PMC11229015; doi:10.1186/s40795-024-00902-5)
Supplement: Supplementary file 5 — Supplementary Material 5. [file 40795_2024_902_MOESM5_ESM.docx]

Table S4. Correlation analysis comparing changes in energy and food group intakes

|  | *r* | *p*-value |
| --- | --- | --- |
| Δ Cereals | 0.489 | <0.001 |
| Δ Potatoes | 0.302 | 0.039 |
| Δ Sugars | 0.104 | 0.487 |
| Δ Pulses | 0.309 | 0.035 |
| Δ Nuts | 0.135 | 0.364 |
| Δ Green and yellow vegetables | 0.053 | 0.723 |
| Δ Other vegetables | 0.105 | 0.483 |
| Δ Fruits | 0.410 | 0.004 |
| Δ Mushrooms | −0.022 | 0.885 |
| Δ Seaweeds | −0.102 | 0.496 |
| Δ Fish and shellfish | 0.399 | 0.005 |
| Δ Meats | 0.537 | <0.001 |
| Δ Eggs | 0.293 | 0.046 |
| Δ Dairy products | 0.395 | 0.006 |
| Δ Animal fats | −0.012 | 0.936 |
| Δ Vegetable oils | 0.460 | 0.001 |
| Δ Confectioneries | 0.496 | <0.001 |
| Δ Alcoholic beverages | 0.006 | 0.967 |
| Δ Non-alcoholic beverages | 0.163 | 0.275 |
| Δ Salt-based seasonings | 0.253 | 0.086 |

*r*, Pearson’s correlation coefficient
