# Supplementary material for: Changes in daily intake of nutrients and foods including confectionery after the initiation of empagliflozin in Japanese patients with type 2 diabetes: a pilot study
Source: BMC Nutr. 2024 Jul 4;10:95. doi: 10.1186/s40795-024-00902-5 (PMC11229015; doi:10.1186/s40795-024-00902-5)
Supplement: Supplementary file 6 — Supplementary Material 6. [file 40795_2024_902_MOESM6_ESM.docx]

Table S5. Principal component analysis results according to change in HbA1c after 4 weeks and changes in energy, nutrient, and food group intakes after 24 weeks

| Principal component | PC1 † | PC2 ‡ | PC3 ¶ |
| --- | --- | --- | --- |
| Eigenvalue  Proportion  Cumulative proportion | 3.441  0.181  0.181 | 2.193  0.115  0.297 | 1.921  0.101  0.398 |
| Factor loading | PC1 | PC2 | PC3 |
| Δ HbA1c #  Δ Energy  Δ Cereals  Δ Potatoes  Δ Pulses  Δ Nuts  Δ Green and yellow vegetables  Δ Other vegetables  Δ Fruits  Δ Mushrooms  Δ Seaweeds  Δ Fish and shellfish  Δ Meats  Δ Eggs  Δ Dairy products  Δ Animal fats  Δ Vegetable oils  Δ Confectioneries  Δ Salt-based seasonings | 0.253  0.848  0.089  0.219  0.483  0.468  0.125  0.479  0.419  0.362  0.219  0.478  −0.011  −0.006  0.511  0.557  0.621  0.364  0.482 | −0.113  −0.412  −0.679  0.268  0.154  −0.300  0.120  −0.061  0.095  −0.250  0.390  0.679  0.196  0.557  0.295  0.359  −0.148  −0.231  −0.145 | 0.263  0.082  0.329  −0.433  −0.122  −0.245  −0.569  −0.448  −0.100  0.285  −0.171  0.070  0.586  0.128  0.299  0.336  0.218  −0.095  −0.454 |

†: 1st principal component

‡: 2nd principal component

¶: 3rd principal component

#: In the factor loadings scatter plots of the principal component analysis, the sign of the change in HbA1c from baseline to this period was reversed so that the relevant factors would cluster in the same direction.
